# Contrasting sulfur isotope signatures in two arid basins separated by the Qilian mountains

**DOI:** 10.1038/s41598-025-02004-z

**Published:** 2025-05-18

**Authors:** Yuxin Hao, Shijiao Niu, Haixia Zhu, Xiying Zhang, Sen Wang, Xiangrui Kong

**Affiliations:** 1 College of Urban and Environmental Sciences, Northwest University, Xi’an, 710127 China; 2 Shaanxi Key Laboratory of Earth Surface System and Environmental Carrying Capacity, Xi’an, 710127 China; 3 Shaanxi Xi’an Urban Ecosystem National Observation and Research Station, National Forestry and Grassland Administration, Xi’an, 710127 China; 4 Key Laboratory of Green and High-end Utilization of Salt Lake Resources, Qinghai Institute of Salt Lakes, Chinese Academy of Sciences, Xining, 810008 China; 5 Qinghai Provincial Key Laboratory of Geology and Environment of Salt Lakes, Qinghai Institute of Salt Lakes, Chinese Academy of Sciences, Xining, 810008 China; 6 Department of Chemistry and Molecular Biology, Atmospheric Science, University of Gothenburg, Gothenburg, 41390 Sweden

**Keywords:** Arid regions, Salt particles, Sulfur isotopes, Qaidam basin, Alxa plateau, Sulfur cycling, Evaporites, Atmospheric chemistry, Environmental chemistry, Geochemistry

## Abstract

Global climate variations have intensified the transformation of salt lakes and saline-alkali playas in arid regions into major sources of saline-alkali dust storms, actively influencing atmospheric circulation and climate dynamics. This study focuses on two geographically close but environmentally distinct regions, the Qaidam Basin and the Alxa Plateau, situated on opposite sides of the Qilian Mountains. Surface salts were collected from both areas and analyzed for ion composition and sulfur isotope characteristics. Results reveal high concentrations of Na^+^ and Cl^−^ in both regions, with Mg^2+^ and SO_4_^2−^ also present, reflecting similarities in ionic composition. The δ^34^S values differ in distribution between the two regions: the Qaidam Basin shows higher and more clustered values (+ 9.11‰ to + 20.23‰), indicating a relatively closed system, while the Alxa Plateau includes lower and occasionally negative values (− 2.30‰ to + 11.43‰), reflecting more variable sulfur inputs and open-system conditions. The study also examines sulfur-to-chloride ratios, which vary significantly across sites in the Alxa Plateau, reflecting complex environmental interactions and diverse sulfur sources. In the Qaidam Basin, sulfur-to-chloride ratios are more stable, suggesting consistent sulfur cycling within a relatively closed environment. Additional analyses of sulfate-to-sodium and sulfate-to-magnesium ratios further emphasize the distinct sulfur sources and processes in each region, with minimal impact from these ions on δ^34^S values. This study provides insights into the contrasting sulfur isotopic and ionic ratios that shape the material sources and evolutionary processes in the Qaidam Basin and Alxa Plateau.

**Supplementary Information:**

The online version contains supplementary material available at 10.1038/s41598-025-02004-z.

## Introduction

Global climate variations have intensified the formation of saline-alkali dust storms from dried saline lakes and playas in arid regions^1^. These storms, now a notable feature of regions such as the Qaidam Basin and Alxa Plateau on opposite sides of the Qilian Mountains in northwest China, highlight broader environmental shifts in arid landscapes. Although these two regions are geographically close, they exhibit distinct environmental characteristics and responses to climate change. The Qaidam Basin, once characterized by saline lakes, is now experiencing rapid desiccation. Low precipitation and high evaporation rates have transformed these lakes into significant dust sources. In contrast, the Alxa Plateau, encompassing vast deserts such as the Badain Jaran and Tengger, has long been an established source of dust^2^. Climate change, driving reduced rainfall and rising temperatures, is intensifying desertification across both regions, resulting in more frequent and far-reaching dust storms. Acting as a climatic boundary, the Qilian Mountains create distinct environmental conditions and material sources for the Qaidam Basin and Alxa Plateau^3^.

The source and transport of salt materials further differentiate these two regions. On the Alxa Plateau, salt materials originate from distant sources like the Mongolian Gobi Desert,^4^ transported through rivers, lakes, and rock fragmentation processes,^5^ with wind dispersing sediments across the plateau^6^. In contrast, the Qaidam Basin primarily relies on local sources, including materials from nearby mountains and hills, with rivers and wind erosion contributing to salt deposits within the basin^7^. These distinct sources of salt materials shape the unique environmental characteristics and landscapes observed in each area. The contrasting climates of the Qaidam Basin and Alxa Plateau present an interesting case for studying the complex dynamics within their saline lake brines. Despite their proximity, these regions are separated by the Qilian Mountains, creating distinct environmental conditions that influence the composition of lake brines and surface salts. Isotopic analysis is particularly useful in this context, as it allows tracing of atomic-level variations in salt materials to reveal their origins, pathways, and transformations. By applying isotopic techniques, this study seeks to reconstruct the history of salt evolution in these lakes, uncovering insights into broader environmental processes and climatic adaptation.

Sulfur exists in several forms, each defined by a distinct isotopic signature. By examining these signatures, it is possible to trace the origins of sulfur in lake brines and evaluate its environmental mobility. This approach is key to understanding the chemical composition and evolution of lakes, especially in regions with diverse climates. Sulfur naturally occurs in four stable isotopes:^32^S,^33^S,^34^S, and^36^S, with isotope compositions typically expressed as δ^34^S values, focusing on the ratio of ^34^S to ^32^S. Sulfate, abundant in terrestrial aquatic systems such as lakes, rivers, and groundwater, plays a vital role in global biogeochemical cycles. Advances in sulfur isotope testing have improved our ability to trace sulfate sources and cycling processes, enhancing our understanding of water runoff, drainage, water quality, and biogeochemical dynamics in aquatic environments^8^.

Sulfur isotope research began in the 1940s and expanded to aquatic environments by the 1960s. Sacks et al.^9^ linked elevated sulfur (sulfate) concentrations in the Florida Aquifer to gypsum dissolution. Michalik et al.^10^ identified atmospheric deposition and sulfide oxidation as major sources of sulfur in the form of sulfate in springs within Polish national parks. Samborska et al.^11^ used sulfur isotopic tracers to estimate the contributions of atmospheric precipitation, sulfide oxidation, and evaporite dissolution in the Upper Silesian Triassic Carbonate aquifer in Poland. Sulfur isotope research in the Qaidam and Alxa regions has primarily concentrated on saline lake brines and evaporite rocks in areas such as Qinghai, Tibet, and Xinjiang. For instance, Fan et al. found that δ^34^S values in Qaidam Basin salt lake brines exceeded those of sulfate-type lakes (11.47‰) and chloride-type lakes (10.45‰)^12^. Li et al.^13^ reviewed δ^34^S values across various water bodies in terrestrial salt-bearing basins, reporting ranges of 0.00‰ to 5.00‰ for atmospheric precipitation, 5.00‰ to 10.00‰ for freshwater, 3.90–6.90‰ for modern salt lakes in Tibet, and 26.46‰ to 54.57‰ for deep oilfield brines in the Qaidam Basin, indicating different levels of bacterial reduction. Further research by Li et al.^14^ on Qarhan Salt Lake revealed δ^34^S values between 6.66‰ and 12.14‰, highlighting the influence of river water recharge on sulfur isotope distribution.

This study aims to investigate the environmentally distinct but geographically close arid regions by analyzing the ionic composition and sulfur isotopic characteristics of surface salts from the Qaidam Basin and Alxa Plateau. By comparing these two regions, this study seeks to elucidate the processes governing sulfur distribution and transformation in saline-alkali environments. The findings will provide a comprehensive framework for understanding the evolution of saline systems in arid regions and their broader implications for dust production, atmospheric chemistry, and climate dynamics.

## Sampling sites

###  Qaidam basin

**Fig. 1:**
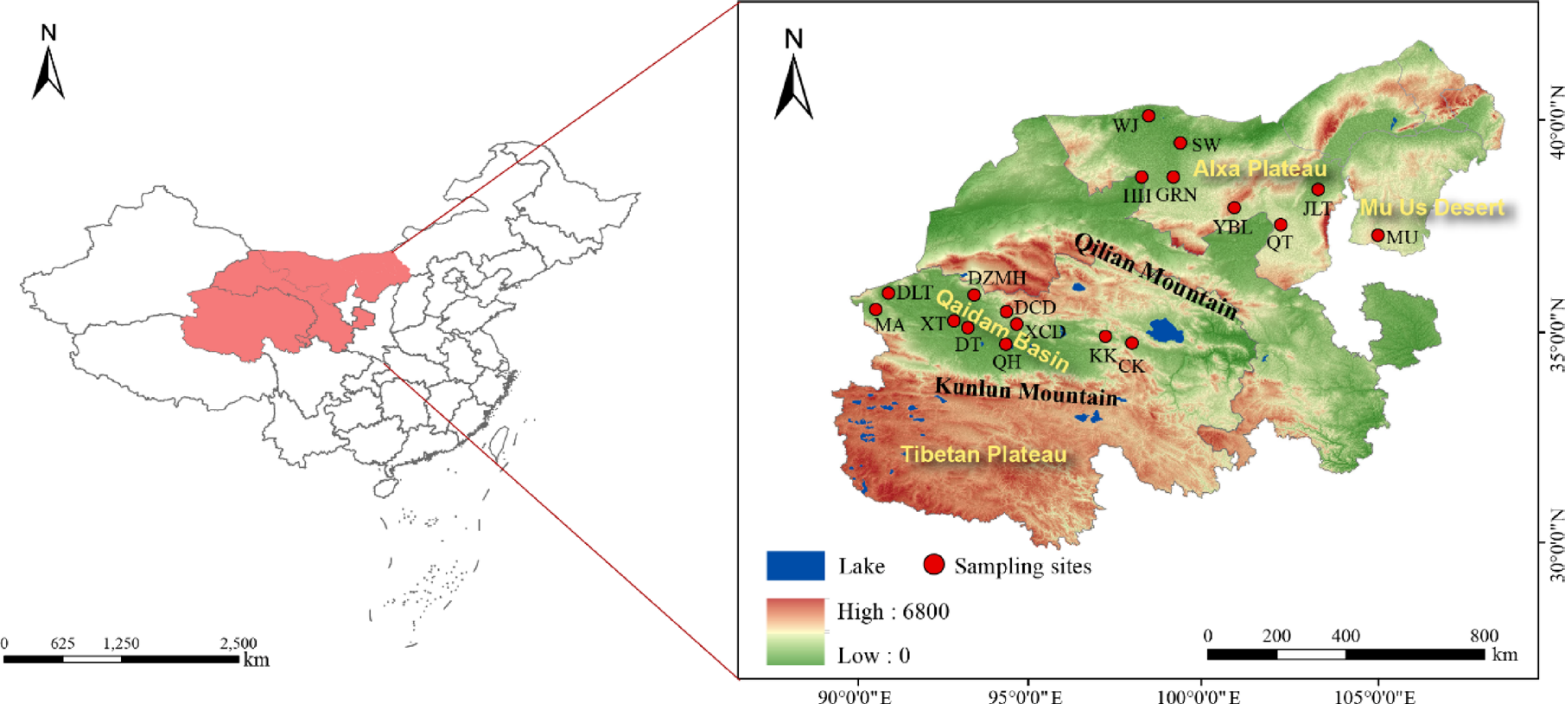
Illustration of the sample collection sites within the Qaidam Basin and Alxa Plateau. Mang’ai Lake (MA), Dalangtan Playa (DLT), Xitaijinar Lake (XT), Dongtaijinar Lake (DT), Dezongmahai Lake (DZMH), Dachaidan Salt Lake (DCD), Xiaochaidan Salt Lake (XCD), Qarhan Salt Lake (QH), Keke Salt Lake (KK), Chaka Salt Lake (CK), West Juyan Lake (WJ), Swan Lake (SW), Heihe River (HH), Gurinai Salt Lake (GRN), Yabulai Salt Lake (YBL), Qingtu Lake (QT), Jilantan Salt Lake (JLT), Mu us Desert (MU). The illustration was created using ArcGIS 10.7, and the DEM digital elevation data used in the map was open-source data obtained from the Geospatial Data Cloud website: http://www.gscloud.cn/.

The Qaidam Basin is located in the northeastern part of the Qinghai-Tibet Plateau, between 90°16′–99°16′ E and 35°00′–39°20′ N (Fig. 1). Surrounded by the Kunlun, Altyn, and Qilian Mountains, this closed plateau basin spans approximately 120,000 km^2^,^15^ wider in the west and narrowing to the east, forming a largely triangular shape^16^. Since the Mesozoic and Cenozoic eras, the basin has been shaped by long-term extensional compression driven by continuous tectonic stress, which has played a critical role in its geological evolution^17^. This tectonic activity caused the subsidence of the ancient Qaidam region, resulting in a series of evolutionary stages, including western depression, central uplift, western uplift, and eastern depression.

The Qaidam Basin has a plateau continental climate marked by aridity, dividing it into two distinct climatic regions: the central arid desert and the surrounding alpine areas^18^. Annual precipitation ranges from 15 mm to 200 mm, with significant regional variability. The average relative humidity fluctuates between 30% and 40%, occasionally dropping below 5%^19^. Average annual temperatures typically remain below 5 °C, with extreme fluctuations and sharp differences between daytime and nighttime temperatures^20^. The interior of the basin is also prone to frequent strong winds, particularly in the western region, which is more vulnerable to severe wind erosion. Over time, these environmental factors have influenced the formation, development, evolution, and eventual desiccation of large ancient lakes within the basin.

The Qaidam Basin is situated at the intersection of three major atmospheric circulation systems: the Westerlies, the Indian Summer Monsoon, and the East Asian Summer Monsoon. As a result, its precipitation sources are complex. However, water vapor in this region primarily originates from the Westerlies and inland recycled moisture. The Westerlies transport a small fraction of ocean-derived water vapor—originating from the Atlantic, Red Sea, Black Sea, Mediterranean, and Caspian Seas—over long distances through Central Asia. During this extended journey, oceanic vapor is progressively depleted, while recycled continental moisture becomes dominant. Consequently, only limited oceanic vapor reaches the Qaidam Basin, and the summer monsoons contribute minimally to local precipitation^21–23^.

### Alxa plateau

The Alxa Plateau, located in the Alxa League of western Inner Mongolia, China, is part of the broader Inner Mongolian Plateau^24^. This inland region, far from the ocean and encircled by mountains, creates a relatively isolated geographical environment. The plateau’s average elevation is about 1,300 m, with a gentle slope from south to north and minimal ground fluctuation. Only a few mountainous areas rise above 2,000 m, with the lowest point near Juyan Lake at 820 m. The landscape features dry, eroded low mountains and hills ranging from 100 to 250 m in height, dividing the plateau into several inland basins^25^.

The Alxa Plateau is a vast arid desert region characterized by extensive areas of quicksand and gobi. It is home to three large deserts: the Badain Jaran Desert, the Tengger Desert, and the Ulan Buhe Desert, which collectively cover nearly one-third of the plateau. The water system in this area is underdeveloped, and the Heihe River and Shiyang River, which originated from the Qilian Mountains, are the only surface water sources^26^. There are over 500 salt and freshwater lakes in the plateau, which are mainly recharged by groundwater that may come from adjacent fault zones in mountainous areas^27^. In addition, some precipitation may reach the groundwater in the shallow groundwater area around the lake, thus becoming the recharge source of desert lakes^26^.

Situated in the hinterland of northwest China and near the Helan Mountains to the west, the Alxa Plateau experiences a typical dry, non-monsoon climate. Its geographical location prevents the summer monsoon from reaching the region, resulting in distinct seasonal variations and significant temperature differences between day and night^28^. The annual average temperature ranges from 2.1 °C to 9.6 °C, with rainfall decreasing from 200 mm in the southeast to less than 40 mm in the northwest, most of which occurs in the summer. The region’s aridity, strong evaporation, and frequent strong winds^29^ make it a major source of sandstorms^30^.

In contrast, the Alxa Plateau lies in central Northwest China within the arid to semi-arid climate zone. Its precipitation is predominantly influenced by continental air masses, with key vapor sources including the Westerlies, polar air masses, inland recycled vapor and, to a lesser extent, summer monsoons^31,32^. Local evapotranspiration from lakes and shallow groundwater further contributes to atmospheric moisture recycling. Additionally, long-range water vapor transport from the Atlantic and Arctic Oceans—via the Black Sea, Caspian Sea, and Aral Sea—can reach the plateau through westerly and polar airflows. Although the Alxa Plateau lies outside the core monsoon influence zone, it may experience weak monsoonal effects during the summer months^32^.

## Methodology and analysis

### Sampling procedure

The sampling process was conducted in three stages:

- September 2020: Brines and crusts were collected from three saline lakes (QH, CK, KK) in the Qaidam Basin.
- May 2021: Surface salt samples, including brine, crust, saline-alkali soil, and salt sand, were collected from seven sites (WJ, SW, HH, QT, YBL, GRN, and JLT) in the Alxa Plateau. Crust samples from the Mu Us Desert (MU) were also collected for comparison.
- May 2022: Brines and crusts were collected from seven saline lakes (MA, DLT, XT, DT, DZMH, DCD, XCD) in the Qaidam Basin.

For sample collection, brine samples were taken from a depth of 2–5 cm below the lake surface, while lakebed salt samples were gathered directly beneath the brine sampling points. Crust, saline-alkali soil, and salt sand samples were collected from dry lakebeds. To ensure representativeness and reliability, a five-point sampling method was used, with samples collected from multiple locations within each site and then combined to capture site-wide characteristics and reduce errors from local heterogeneity. These samples were subsequently analyzed to investigate the sources and evolutionary processes of surface salts across the different regions.

### Sample preparation and storage

All collected samples were stored in polyethylene bottles. To ensure their stability and purity, the samples were sealed with Parafilm membranes and kept at 4 °C for preservation. Solid samples were dissolved in ultrapure water to prepare saturated solutions, and the pH of these solutions was measured using a pH meter (FE28-Standard, Mettler Toledo, China). Saturated salt solutions from various sample sites were allowed to settle for 24 h, after which the supernatant was collected. A further filtration process was conducted using a 0.22 μm pore syringe filter to remove any remaining insoluble particles^33^. To ensure compatibility with ion chromatography (IC) detection limits, all sample solutions were diluted 1500 times before analysis.

### Ion chromatography

A Thermo Fisher Scientific IC system (DX-600) was used to simultaneously measure the concentrations of cations (K^+^, Na^+^, Mg^2+^, Ca^2+^, and NH_4_^+^) and anions (Cl^−^, SO_4_^2−^, NO_3_^−^, NO^2−^, and F^−^) in the sample solutions, with the measurement error controlled within 5%. For cation analysis, a CS12AIC column (Dionex Ion Pac, Thermo Fisher Scientific) with a 30 mmol/L methanesulfonic acid eluent was used, while an AS11-HCIC column (Dionex Ion Pac, Thermo Fisher Scientific) with a 20 mmol/L KOH eluent was used for anion determination. Prior to sample analysis, a blank experiment using pure water was conducted to establish a baseline. The resulting value from this control experiment was subtracted from the sample data to eliminate potential contaminants from the water or filter, ensuring accuracy and purity. Additionally, the saturated salt solutions were diluted 20 times, and the concentrations of CO_3_^2−^ and HCO_3_^−^ in brines and salt solutions were determined through acid-base titration analysis. All samples underwent duplicate analyses, demonstrating excellent reproducibility.

### 3Isotopic analysis

Sulfur isotope measurements were performed at the National Laboratory of Biogeology and Environmental Geology, China University of Geosciences. Five grams of solid salt samples were dissolved in 10 ml of ultrapure water. The salt solutions from various salt lakes were first filtered through qualitative filter paper with a pore size of less than 20 μm and then passed through 0.22 μm filtration membranes. For each sample, 3 ml of the filtered solution was taken, and 2 to 3 drops of hydrochloric acid (1:1) were added. The mixture was thoroughly shaken and allowed to stand for 30 min, after which the pH was checked using test paper to ensure it was less than three.

To facilitate complete mixing of sulfate and barium ions, 5 ml of a 250 g/L barium chloride solution was added to the salt solutions and shaken for approximately one hour. After standing for 24 h, a white precipitate formed at the bottom of the bottle. The solution was filtered through a cellulose acetate membrane using a suction filter pump and repeatedly washed with deionized water to obtain pure barium sulfate. The filter membranes were placed into clean crucibles and calcined in an 800 °C muffle furnace for two hours, burning off the filter membranes and leaving pure barium sulfate powder.

The collected barium sulfate was mixed with an excess of vanadium pentoxide (V_2_O_5_) and subjected to online combustion to decompose the sample and generate sulfur dioxide (SO_2_) gas for isotopic analysis. The resulting SO₂ was measured using an isotope ratio mass spectrometer (Delta V Plus, Thermo Fisher Scientific, USA) coupled with a Flash elemental analyzer to determine the sulfur isotope composition of the carbonate-associated sulfate (δ^34^S_CAS_). Sulfur isotope compositions are expressed in standard δ-notation as per mil (‰) deviation from the V-CDT international standard, with an analytical error of 0.2‰ (1σ) calculated from replicate analyses of IAEA standards (GBW04415, IAEA-SO-5, IAEA-SO-6)^34^. The δ^34^S (‰) value is calculated as follows:

1 $$\:{{\updelta\:}}^{34}\text{S}=\frac{{\left({}^{34}S/{}^{32}S\right)}_{Sample}-{\left({}^{34}S/{}^{32}S\right)}_{Standard}}{{\left({}^{34}S/{}^{32}S\right)}_{Standard}}\times\:1000$$

## Results

### Chemical compositions

#### Salt crusts

**Fig. 2:**
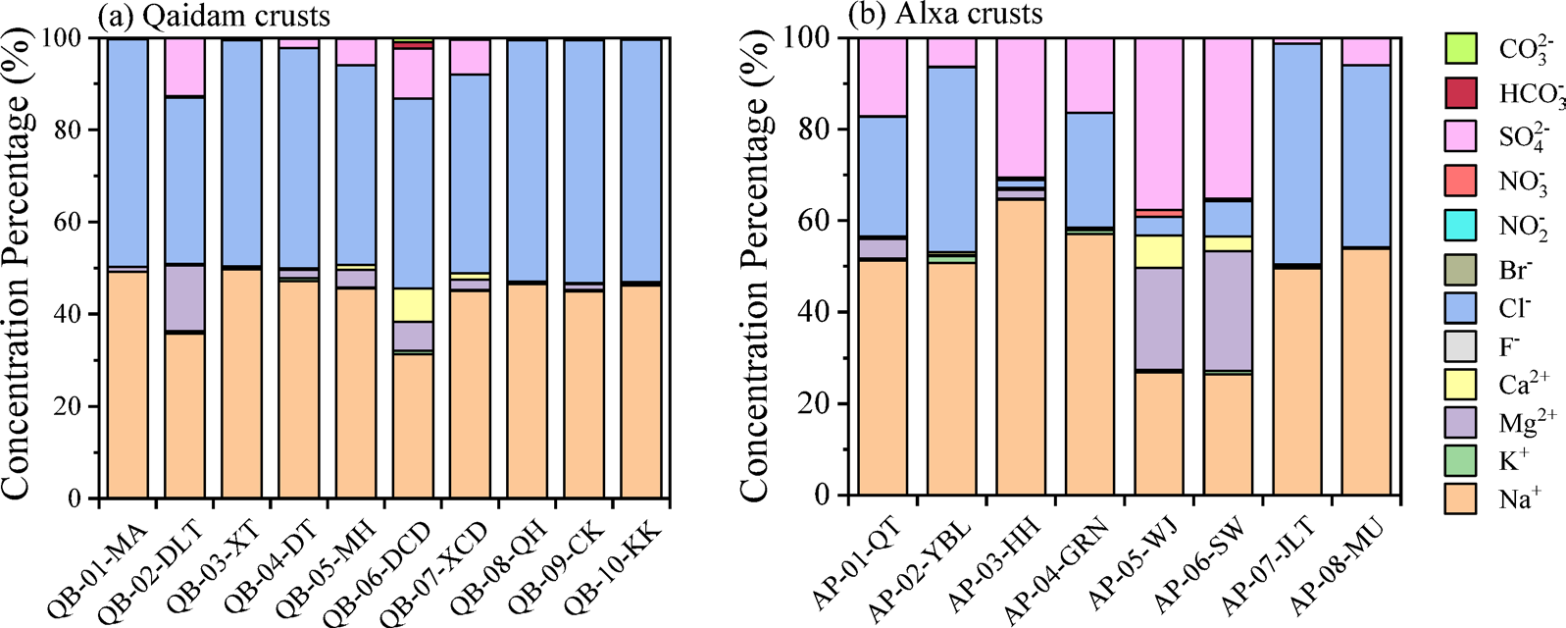
Normalized ionic molar fractions of salt crusts in (**a**) Qaidam Basin, (**b**) Alxa Plateau.

The ionic compositions of natural samples were measured using IC, analyzing the concentrations of main ions from brines and salt crusts in different saline lakes in the Qaidam Basin and Alxa Plateau. Figure 2 shows that the ion compositions of crust salts in the same region showed high similarity. The crusts from each saline lake in the Qaidam Basin (Fig. 2a) are primarily composed of Na^+^ and Cl^−^, indicating that halite is the main component. There is no clear correlation between crust formation and geographical location or lake type; however, variations in precipitation and lake water levels may influence salt formation at the soluble interface, with salts accumulating between the brine-lakebed and crust-air interface^14,35^. In some lakes, specific ions are present in notably higher concentrations. The crusts from DLT, DCD, and DZMH show elevated levels of Mg^2+^, likely due to the geological environment of these sites, such as that DLT is located in the magnesium-rich Liangzhong deposit. Additionally, DCD displays a higher concentration of Ca^2+^, which may result from an influx of sand particles and gypsum, especially in areas near the desert where fewer other salts are present. Furthermore, the crusts from DLT, DCD, XCD, DZMH, and DT exhibit relatively high SO_4_^2−^ levels, suggesting that sulfate salts are more prominent in these locations. Despite these variations, NaCl remains the primary component across most sites in the Qaidam Basin.

For the Alxa Plateau crusts, as shown in Fig. 2b, Na^+^ is the major ion, though NaCl is in general less dominant here than in the Qaidam Basin. The crusts from QT, YBL, GRN, JLT, and MU exhibit high levels of Cl^−^ as the dominant anion, whereas SO_4_^2−^ is notably high in HH, WJ, and SW. It is likely that these crusts contain a mix of NaCl and Na_2_SO_4_. In the WJ and SW crusts, Mg^2+^ is present at comparable levels to Na^+^, distinguishing these sites from others in the region. Additionally, these crusts show elevated levels of Ca^2+^, which may be sourced from desert sands transported into the lake regions by wind. The Heihe River, which flows through these sites, may also contribute to this unique ionic composition by carrying materials from the surrounding desert^36^. K^+^ is measurable in the crust from YBL, which may result from localized agricultural activities introducing potassium into the environment. This observation highlights the interaction between natural desert inputs, anthropogenic activities, and surface salt compositions, providing insights for further studies on the environmental and geological factors affecting surface salts in arid regions.

#### Brines

**Fig. 3:**
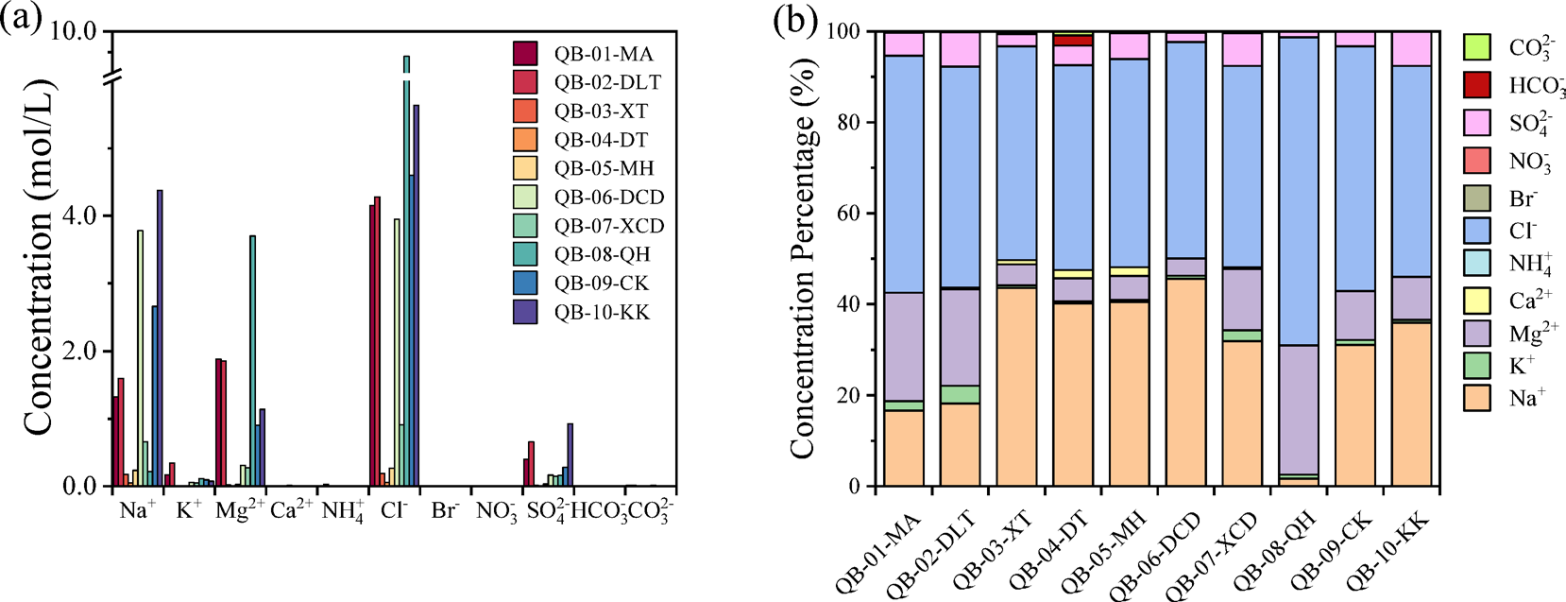
(**a**) Ionic molar concentrations of Qaidam Basin brine samples, represented by bars of different colors, with each color corresponding to a specific salt lake. (**b**) Normalized ionic molar fractions of brines from the Qaidam Basin.

A comparison of ion concentrations in brine samples from various saline lakes in the Qaidam Basin reveals substantial differences in brine composition (Fig. 3). The salt lakes in the Qaidam Basin are a rich resource, containing high concentrations of various ions that are valuable for mineral extraction and industrial applications. The elevated ion concentrations in lakes MA, DLT, QH, CK, and KK across the eastern and western regions of the Qaidam Basin are likely driven by high evaporation rates, inflows from mineral-rich sources such as groundwater and oilfields, and proximity to geologically enriched deposits, which together contribute to significant mineral accumulation. In contrast, other lakes with lower ion concentrations experience less evaporation, different water sources, and possibly more rainfall, resulting in a lower degree of mineralization.

Salt-forming processes in the western Qaidam Basin began earlier than in the eastern region, with Dalangtan Playa nearly reaching the final stage of its evolution. Due to lower precipitation levels in the western basin, brines there evaporate more readily. The brines in MA and DLT are mainly replenished by groundwater and oilfield water, which contributes to their higher mineralization and elevated ion concentrations.

In the eastern region of the Qaidam Basin, hot springs are prevalent. Hot spring water around QH, CK, and KK acts as a recharge source, increasing the ion concentrations in these saline lake brines. Meanwhile, the central saline lakes are situated at lower altitudes. The hydrological recharge system in the southern part of the basin primarily originates from the Kunlun Mountains, feeding lakes such as XT, DT, and QH. In the north, lakes are supplemented by runoff from the Qilian Mountains, including DZMH, DCD, and XCD^37^. As a result, ion concentrations in the brines of these saline lakes tend to be generally lower.

Brines exhibit distinct ionic compositions compared to salt crusts. Brines are typically rich in Cl^−^, Na^+^, and Mg^2+^ compared to solid salts and contain higher levels of HCO_3_^−^ and CO_3_^2−^ (Fig. 3), possibly due to the absorption of CO_2_ from the surrounding environment. Notably, QH shows a unique composition among the saline lakes, with significantly higher concentrations of Mg^2+^ and K^+^. This is likely due to the abundance of sodium, potassium, and magnesium resources in the area, which aligns with QH being home to the largest potassium-magnesium salt deposit in China^38^.

### pH

**Fig. 4:**
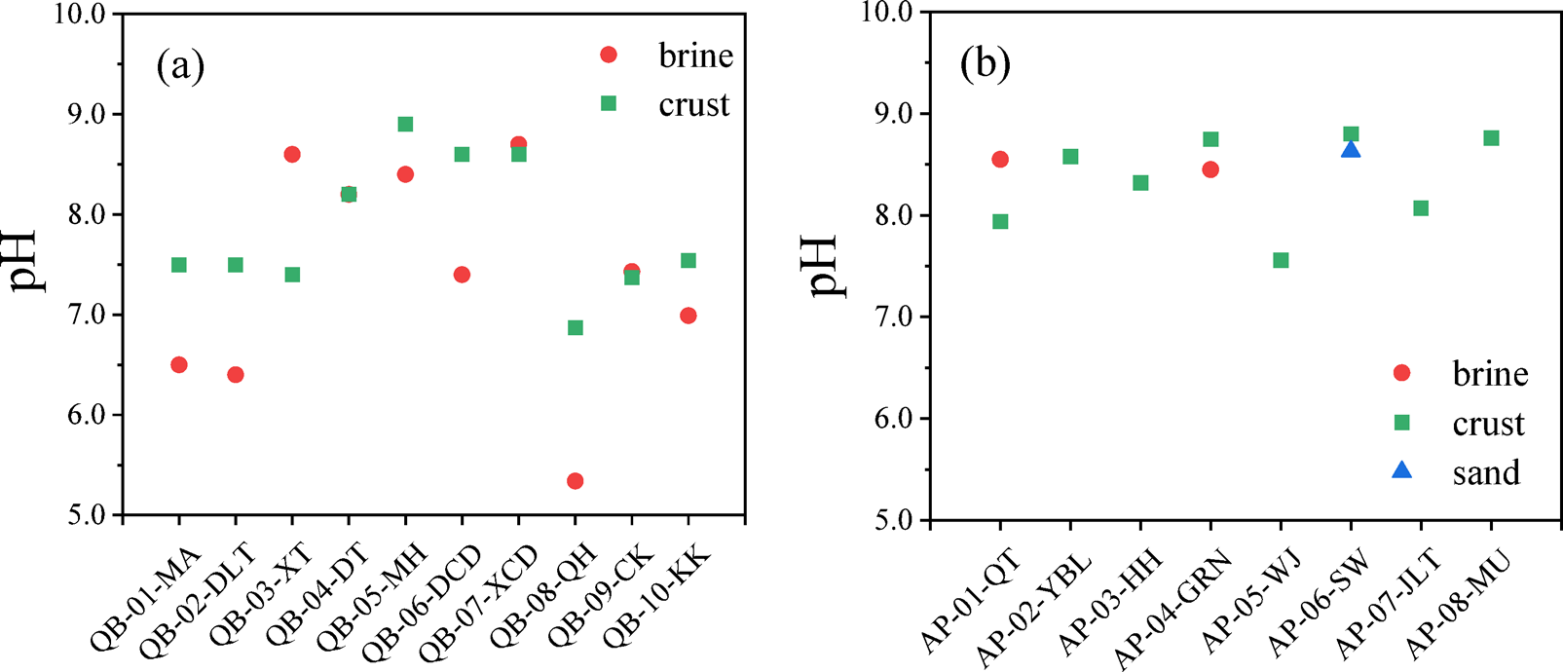
pH values of various surface salt types in (**a**) the Qaidam Basin and (**b**) the Alxa Plateau. The pH measurements were taken from saturated solutions of crust and sand samples.

The pH of saline lake brines (Fig. 4) is influenced by their chemical composition, with higher mineralization generally leading to lower pH. Among lake types, pH decreases in the order of carbonate-type, sulfate-type, and chloride-type lakes^39^. In the Qaidam Basin, brine pH is generally lower than the crust salt saturated solutions, except in lakes XT and XCD. Acidic brines are observed in lakes MA, DLT, and QH (pH as low as 5.34), while lakes DCD, CK, and KK have neutral to slightly alkaline brines (pH 7.00–7.43). Surface salts from XT, DT, DZMH, and XCD exhibit alkaline pH values above 8.20.

Lake classifications align with pH trends, with chloride-type lakes (e.g., QH) having lower pH than magnesium sulfate or sodium sulfate lakes^40–42^. Salt crusts, however, show more stable pH due to the formation of chemically stable crystals, preserving records of past brine environments and climate. On the Alxa Plateau, crust pH ranges from 7.56 to 8.76, similar to Qaidam crusts, with minimal variation across salt types. Comparing both regions, the lowest pH values occur in Qaidam Basin brines, reflecting distinct geochemical environments.

### Sulfur isotopic ratio

**Fig. 5:**
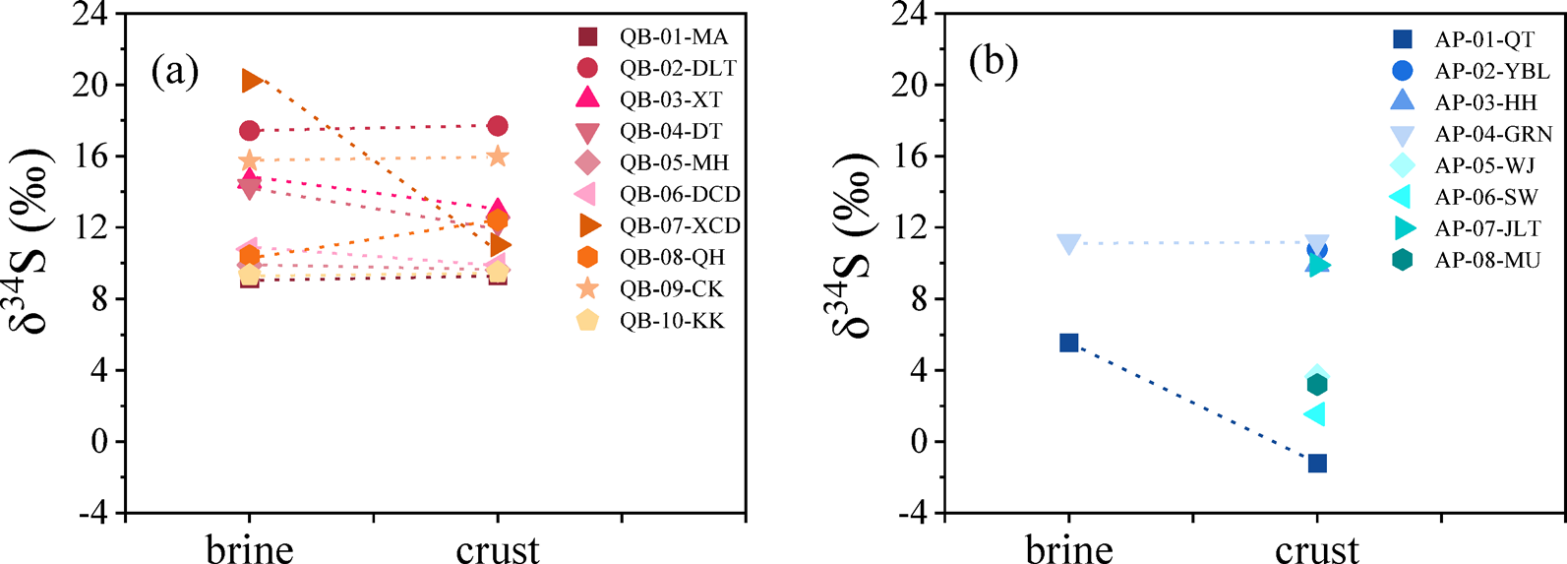
The δ^34^S values of different types of surface salts in (**a**) the Qaidam Basin, (**b**) Alxa Plateau and Mu Us Desert. Red represents Qaidam, blue represents Alxa, and green represents Mu Us. Different sampling sites are distinguished by color depth. The dashed line connects samples from the same salt lake.

Sulfur isotope measurements provide valuable insights into the origins and environmental interactions of surface salts. In the Qaidam Basin, the δ^34^S values of surface salts collected from most sampling sites vary between + 9.11‰ and + 20.23‰, remaining relatively consistent across different sample types, with the exception of XCD (Fig. 5a). This consistency indicates that surface salts within the same location likely share a common origin, and any structural differences among the salt samples are likely due to local dissolution and evaporation processes. This observation aligns with previous research, which has shown minimal sulfur isotope fractionation between evaporites and their corresponding brines in arid regions^43,44^. For surface salts from MA, DZMH, DCD, and KK, δ^34^S values (+ 9.11‰ to + 10.78‰) are in line with reported values for freshwater, brine, and atmospheric precipitation in northern China^14^. This suggests strong interaction between these four saline lakes and the surrounding environment, with brines likely supplemented by surface water and atmospheric precipitation. These results highlight how the sulfur isotope values of surface salts can be influenced by local environmental material exchanges.

Interestingly, there is a notable difference in δ^34^S values between the brines and crusts of XCD. Several factors may account for these differences, including variations in material sources, isotopic fractionation, and bacterial sulfate reduction (BSR)^45–47^. Given that the XCD sampling site is located near a highway, it is hypothesized that the samples may have been affected by anthropogenic sources.

In the Alxa Plateau, δ^34^S values for YBL, HH, GRN, and JLT range from + 9.88‰ to + 11.20‰ (Fig. 5b), consistent with δ^34^S values for freshwater, brine, and atmospheric precipitation in northern China^14^. These values indicate strong material exchange between these areas and the surrounding environment, with the water bodies likely recharged by alpine glacier melt and atmospheric precipitation. However, the δ^34^S values for samples from QT, WJ, SW, and MU are relatively low, particularly the crust from QT and the salt sand from SW, which show negative δ^34^S values. These lower values may result from specific sulfur sources or processes, such as anthropogenic pollution from industrial emissions,^48–50^ microbial sulfur reduction in low-oxygen environments, or geological sources like sulfide minerals with inherently low δ^34^S values. Overall, sulfur isotope values in the Alxa Plateau and Mu Us Desert are notably lower than those observed in the Qaidam Basin, reflecting distinct environmental and geological influences across the regions.

### δ^34^S and ionic ratios

**Fig. 6:**
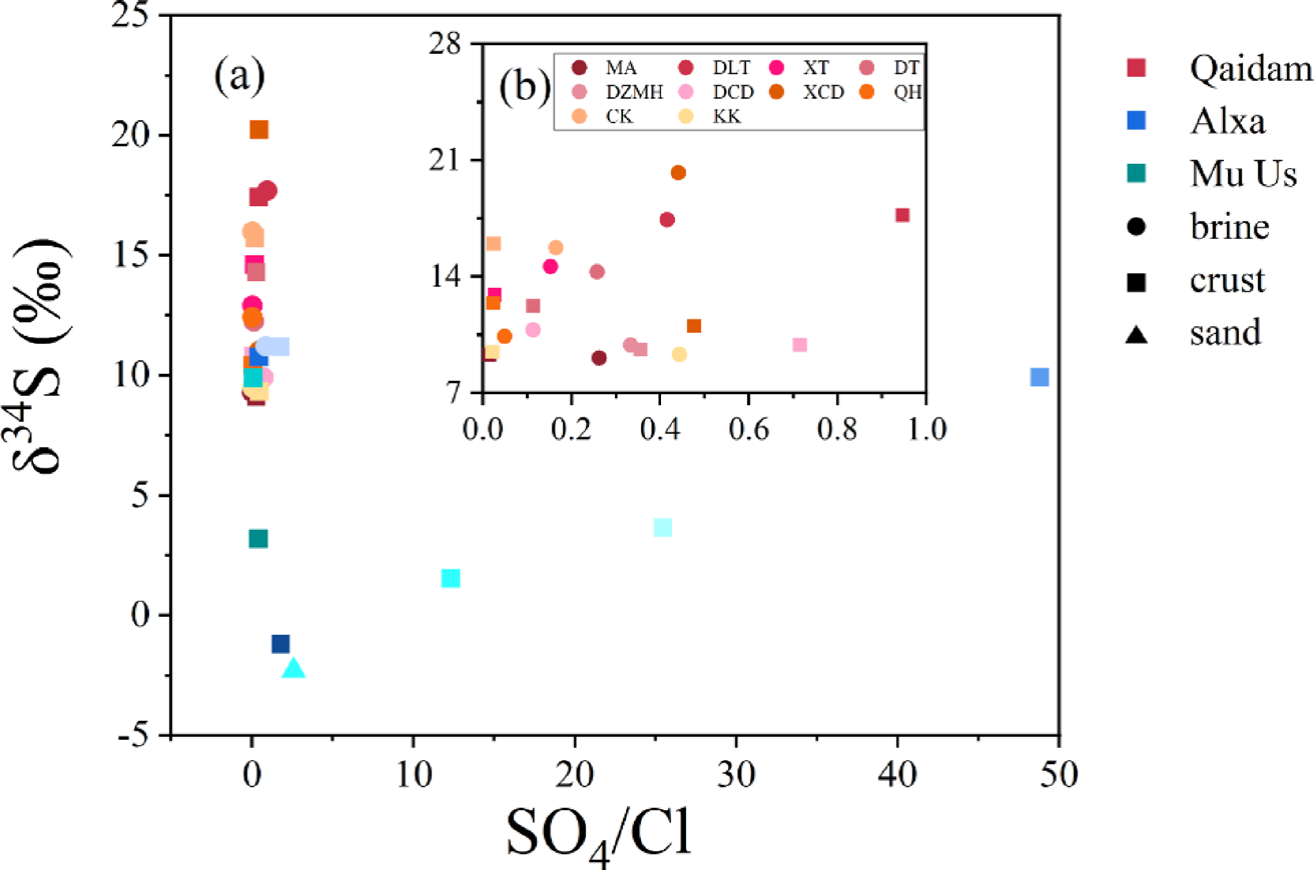
(**a**) The δ^34^S values of different sampling sites of surface salts as a function of SO_4_/Cl ratio in the Qaidam Basin, Alxa Plateau and Mu Us Desert, (**b**) different salt samples in Qaidam Basin are amplified. Red represents Qaidam, blue represents Alxa, and green represents Mu Us. Circles represent brine, squares represent crust, and triangles represent sand. Different sampling sites are distinguished by color depth.

The relationship between δ^34^S values and SO_4_/Cl ratios provides valuable insights into sulfur cycling and bacterial sulfate reduction (BSR) processes,^51^ making it an interesting metric for environmental studies. Analysis of δ^34^S values alongside SO₄/Cl ratios (Fig. 6) reveals considerable variation in the SO_4_/Cl ratios of surface salts across different sites in the Alxa Plateau (Fig. 6a). In the Qaidam Basin, however, variations in the SO_4_/Cl ratio of surface salts from the same saline lake did not significantly affect δ^34^S values, and no clear correlation was observed between δ^34^S values and SO_4_/Cl ratios for most Alxa Plateau salt samples (Fig. 6b).

The relationship between δ^34^S and SO_4_/Cl ratios is a widely recognized proxy for evaluating BSR, as a negative correlation typically indicates active BSR processes. During BSR, microbes preferentially reduce the lighter sulfur isotope (^32^S), leaving the residual sulfate enriched in ^34^S and thus increasing δ^34^S values in brines and precipitated salts. Additionally, sulfate released from pore waters beneath the sediment–water interface following BSR can further influence the isotopic composition^52^. In this study, however, we do not observe a clear negative correlation between δ^34^S and SO_4_/Cl ratios, suggesting that BSR is likely not the dominant control on sulfur isotopic variation. Instead, the observed δ^34^S values are more plausibly attributed to mixing of sulfur from multiple sources, including atmospheric deposition, riverine input, and groundwater recharge. Elevated δ^34^S values may also reflect limited external exchange, consistent with closed-system conditions in some saline lakes. Moreover, spatial variations in δ^34^S values provide a useful framework for distinguishing between natural and anthropogenic sulfate sources, offering important insights into the origin of sulfate aerosols and associated SO_2_ in arid environments^53,54^.

The relationships between δ^34^S values and various ion ratios—SO_4_/Na, SO_4_/Mg, and SO_4_/total ion—were analyzed to understand sulfate sources and processes in the Qaidam Basin and Alxa Plateau. For the SO_4_/Na ratio (Figure S1), no significant correlation with δ^34^S values was observed in either region, suggesting that Na⁺ concentrations do not directly affect sulfur isotope composition. Similarly, SO_4_/Mg ratios (Figure S2) showed wide variation, particularly in the Alxa Plateau, but no clear relationship with δ^34^S values was found, indicating that Mg^2+^ also has minimal impact on sulfur isotope composition in these regions.

The analysis of SO₄/total ion ratios (Figure S3) revealed differences across samples, especially in the Alxa Plateau, but these fluctuations did not significantly influence δ^34^S values in the Qaidam Basin. This suggests that variations in sulfate relative to total ion content do not strongly affect sulfur isotope composition. Further analysis of the correlation between δ^34^S values and these ion ratios (Figure S4) indicated that, overall, there are no significant correlations across all ion ratios. This lack of correlation implies that δ^34^S values are more likely influenced by factors such as sulfur source mixing and local environmental conditions, rather than by any specific ion concentration.

## Discussions

**Fig. 7:**
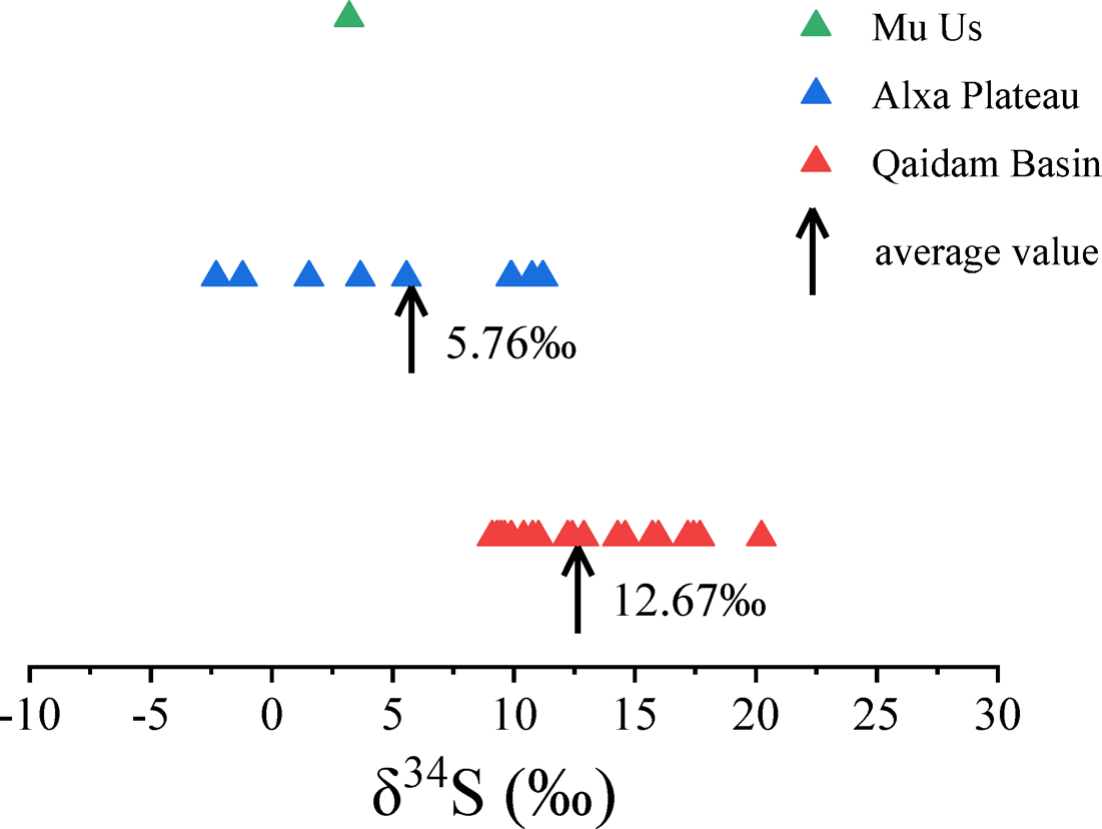
The distribution of δ^34^S values in Qaidam Basin, Alxa Plateau and Mu Us Desert.

The Qaidam Basin and Alxa Plateau lie on opposite sides of the Qilian Mountains, which create a significant climatic barrier, resulting in distinct terrain and climate conditions in each region. As shown in Fig. 7, the δ^34^S values of surface salts in the Qaidam Basin are relatively high and consistent, ranging from + 9.11‰ to + 20.23‰, with an average of + 12.67‰. In contrast, the δ^34^S values in the Alxa Plateau are notably lower and more variable, from − 2.30‰ to + 11.43‰, averaging + 5.76‰. Some Alxa Plateau samples, such as the crust from QT and the salt sand from SW, even exhibit negative δ^34^S values.

These distinct δ^34^S characteristics likely result from different environmental interactions and material exchanges. In the Alxa Plateau, lower and variable δ^34^S values reflect an open system with significant material exchange, including inputs from river inflows, atmospheric precipitation, and groundwater recharge. This high degree of interaction allows for diverse sulfur sources, including dust and sediment transported from Central Asia and Mongolia. These inputs, combined with dynamic processes such as lake water evaporation and crystallization, produce a broad range of δ^34^S values, indicative of both local and distant sulfur sources.

In contrast, the Qaidam Basin exhibits higher δ^34^S values, suggesting a more closed environment with limited external sulfur sources. This isolation, driven by restricted hydrological inputs and high evaporation rates, promotes isotopic enrichment over time, with ^34^S accumulating as lighter ^32^S isotopes escape in volatile compounds^55^. This fractionation process is enhanced by arid conditions and contributes to the higher δ^34^S values observed, especially in areas where evaporation and crystallization are dominant.

**Fig. 8:**
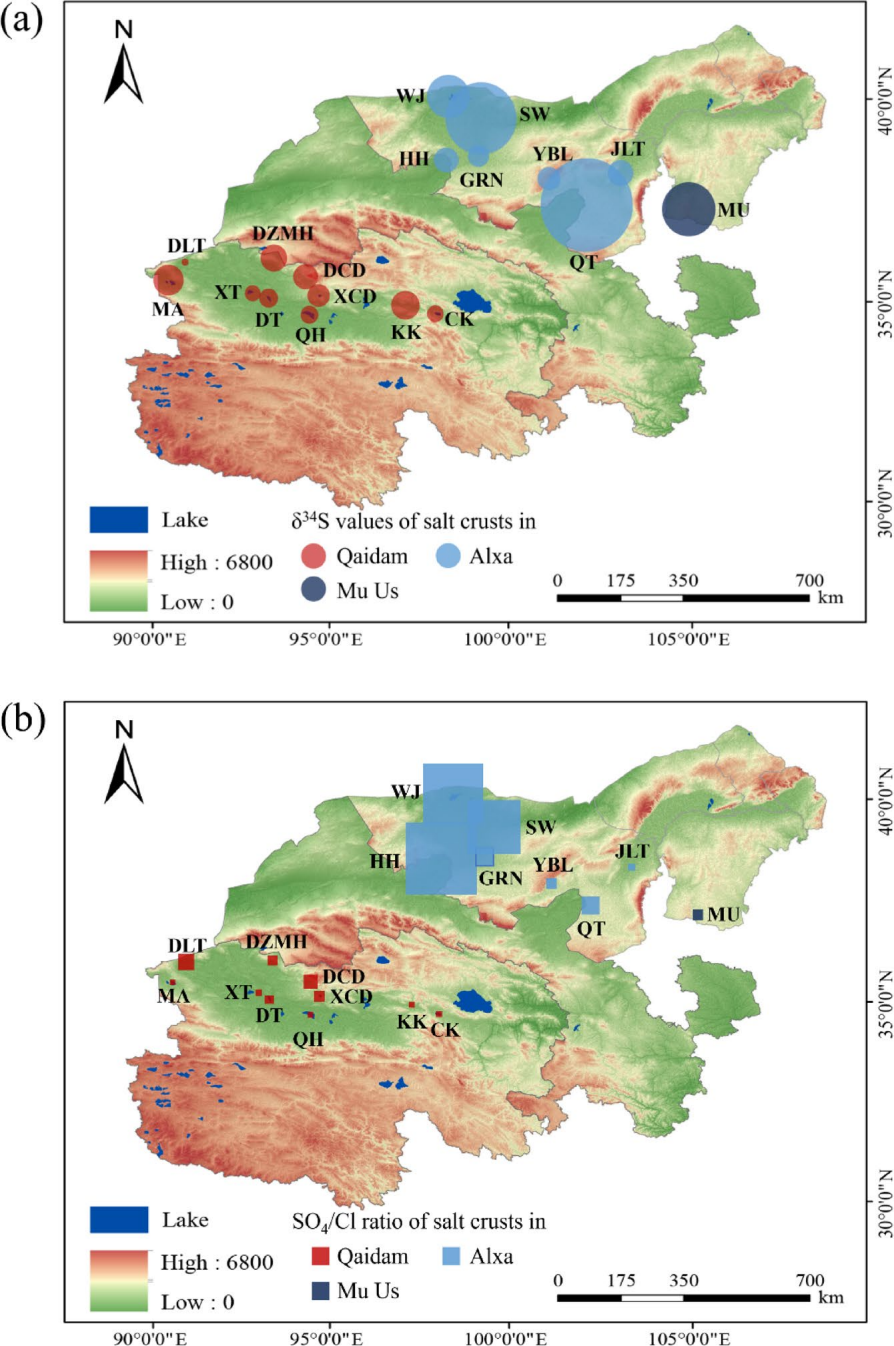
(**a**) The δ^34^S values of different types of surface salts, and (**b**) the δ^34^S values of different types of surface salts as a function of SO_4_/Cl ratio in Qaidam Basin, Alxa Plateau and Mu Us Desert. Two regions are distinguished by different colors. The circle diameter is proportional to the exponential of the negative δ^34^S value, calculated as diameter = exp(-δ^34^S), meaning that diameter decreases with increasing δ^34^S, including negative values. In (b), the diagonal of each square corresponds to the SO_4_/Cl ratio. The illustration was created using ArcGIS 10.7, and the DEM digital elevation data used in the map was open-source data obtained from the Geospatial Data Cloud website: http://www.gscloud.cn/.

In addition to δ^34^S values, SO_4_/Cl ratios offer further insights into the sulfur cycling processes within these regions. As shown in the map in Fig. 8, δ^34^S and SO_4_/Cl ratios vary across the sampled areas in both the Qaidam Basin and Alxa Plateau. In the Qaidam Basin, SO_4_/Cl ratios tend to remain relatively stable across different sites, reflecting a more closed system where limited external input and consistent local processes maintain the sulfur balance. In the Alxa Plateau, however, SO_4_/Cl ratios exhibit more variability, similar to the δ^34^S values, which indicates a more open system. This variability suggests that diverse sources, such as river inflows, atmospheric deposition, and regional dust inputs, influence sulfate levels in the Alxa Plateau. The lack of a strong correlation between SO₄/Cl ratios and δ^34^S values in the Alxa Plateau further supports the presence of multiple sulfur sources and complex material exchanges in this region.

The differences in material sources also contribute to these distinct isotopic signatures and SO₄/Cl patterns. In the Qaidam Basin, primary material sources include detrital sediments from surrounding mountains and hills, along with river water and internal sedimentation processes within the basin. Exposed gypsum-bearing evaporite formations are present within the Qaidam Basin and nearby highlands. These deposits were formed under arid climatic conditions during periods of strong evaporation in lacustrine environments. The gypsum layers, along with associated evaporites, are part of sedimentary sequences that span from the Miocene to the Pleistocene. In addition to evaporites, the regional stratigraphy includes conglomerates, siltstones, minor mudstones, and eolian sediments, all of which contribute detrital material to the basin via fluvial and aeolian processes^56^. These materials are mainly controlled by the region geology and influenced by wind action, which transports fine particles and further isolates the system from broader environmental interactions^57,58^. This relative isolation enhances the closed-system dynamics of the Qaidam Basin, leading to higher δ^34^S values and stable SO₄/Cl ratios as sulfur isotopes and sulfate levels accumulate over time with minimal external input.

On the other hand, the Alxa Plateau is characterized by a much wider range of material sources. The flat northern part of the region allows for the inflow of materials from both local and distant origins, including those transported from the Mongolian region. Previous studies suggest that the Alxa Plateau’s materials are derived from the weathering and denudation of Mesozoic and Cenozoic sandstones, sandy conglomerates, and clastic rocks^59,60^. Additionally, extensive lake sediments from dry lake beds in the western and northwestern parts of the region further contribute to the composition of surface salts^61^. The Alxa Plateau lies between the Gobi Desert to the north and the northeastern margin of the Tibetan Plateau to the south, giving rise to a complex geomorphological and sedimentary environment. The region is influenced by both external and local material sources that shape the composition of surface salts. Long-range transported dust from Central Asia and the Mongolian Gobi Desert, carried by the Westerlies and Siberian High-pressure systems, represents a major external sulfur source^62^. This fine particulate matter is deposited across the plateau, enriching lakebeds with sulfate-bearing minerals. In addition to far-source inputs, near-source materials also play an important role. Fluvial transport from surrounding mountain ranges contributes clastic debris and weathered rock particles to local lakes and playas. These materials, derived from Mesozoic–Cenozoic sandstones, conglomerates, and evaporitic sediments, add further complexity to the sulfur isotope composition^63,64^. The combined influence of distant dust and local geological inputs leads to a wide range of δ^34^S values and variable SO_4_/Cl ratios across Alxa sampling sites, reflecting a more open and dynamic sulfur cycling system compared to the Qaidam Basin.

The contrasting geological formations, climatic conditions, and material sources in these two arid regions play critical roles in shaping both the sulfur isotope compositions and SO_4_/Cl ratios observed in the surface salts. The Qaidam Basin, with its enclosed environment, shows a gradual accumulation of heavier sulfur isotopes and stable sulfate-to-chloride ratios, while the Alxa Plateau, with its dynamic exchange processes and broader range of material interactions, exhibits more variable δ^34^S values and SO₄/Cl ratios. These differences not only highlight the distinct environmental processes at work in these regions but also provide insights into the broader mechanisms of sulfur cycling in arid and semi-arid environments.

##  Conclusions

This study highlights the distinct sulfur isotopic and chemical compositions of surface salts in the Qaidam Basin and Alxa Plateau, two arid regions in northwest China. Despite their proximity, the regions display different δ^34^S values due to contrasting environmental processes, material sources, and climate conditions. In the Qaidam Basin, high and concentrated δ^34^S values indicate a closed system with limited external exchange. Sulfur here is largely derived from local geological materials and influenced by high evaporation rates, leading to isotopic enrichment of ^34^S. In contrast, the Alxa Plateau shows a broader range of δ^34^S values, suggesting an open system with diverse material sources, including river inflows, atmospheric precipitation, and long-range dust transport. The dynamic material exchange in this region results in more variable sulfur isotopic compositions. These differences underscore the role of local environmental factors and material exchanges in shaping sulfur isotope variations in arid regions, providing insights into sulfur cycling and material evolution in such environments. The findings offer a foundation for understanding similar processes in other arid landscapes.

## Electronic supplementary material

Below is the link to the electronic supplementary material.

Supplementary Material 1.

## Data Availability

The datasets used and/or analysed during the current study available from the corresponding author on reasonable request.
