# Supplementary material for: Contrasting sulfur isotope signatures in two arid basins separated by the Qilian mountains
Source: Sci Rep. 2025 May 18;15:17251. doi: 10.1038/s41598-025-02004-z (PMC12086233; doi:10.1038/s41598-025-02004-z)
Supplement: Supplementary file 1 — Supplementary Material 1. [file 41598_2025_2004_MOESM1_ESM.docx]

**Supporting Information**

*for*

**Contrasting Sulfur Isotope Signatures in Two Arid Basins Separated by the Qilian Mountains**

Yuxin Hao^1,2,3^, Shijiao Niu^1,2,3^, Haixia Zhu^4,5^, Xiying Zhang^4,5^, Sen Wang^1,2,3*^, Xiangrui Kong^6*^

*^1^College of Urban and Environmental Sciences, Northwest University, Xi'an 710127, China*

*^2^Shaanxi Key Laboratory of Earth Surface System and Environmental Carrying Capacity, Xi'an, 710127, China*

*^3^Shaanxi Xi'an Urban Ecosystem National Observation and Research Station, National Forestry and Grassland Administration, Xi'an 710127, China*

*^4^Key Laboratory of Green and High-end Utilization of Salt Lake Resources, Qinghai Institute of Salt Lakes, Chinese Academy of Sciences, Xining, 810008, China.*

*^5^Qinghai Provincial Key Laboratory of Geology and Environment of Salt Lakes, Qinghai Institute of Salt Lakes, Chinese Academy of Sciences, Xining, 810008, China.*

*^6^Department of Chemistry and Molecular Biology, Atmospheric Science, University of Gothenburg, SE-41390 Gothenburg, Sweden*

Corresponding authors: kongx@chem.gu.se (X.K.); wangsen@nwu.edu.cn (S.W.)

**Table S1.** Sample type

| Sampling sites | Sample number | latitude and longitude | Sample type |
| --- | --- | --- | --- |
| Mang’ai saline lake | 1 | 90.99580765, 38.07600088 | Brine |
|  | 2 | 90.99580765, 38.07600088 | Lakebed salt |
|  | 3 | 90.99580765, 38.07600088 | Crust |
| Dalangtan playa | 4 | 91.45936310, 38.55716825 | Hydrated salt |
|  | 5 | 91.45936310, 38.55716825 | Hydrated salt |
|  | 6 | 91.45936310, 38.55716825 | Hydrated salt |
|  | 7 | 91.45936310, 38.55716825 | Hydrated salt |
|  | 8 | 91.39273167, 38.53790267 | Brine |
|  | 9 |  | Lakebed salt |
|  | 10 |  | Crust |
|  | 11 | 91.31278574, 38.48132219 | Glauber salt |
| West Taijinar Lake | 12 | 93.45488906, 37.73234612 | Brine |
|  | 13 | 93.45488906, 37.73234612 | Crust |
| East Taijinar Lake | 14 | 93.79405975, 37.59872814 | Brine |
|  | 15 | 93.79405975, 37.59872814 | Crust |
| Dezongmahai lake | 16 | 94.29568648, 38.27253271 | Brine |
|  | 17 | 94.29568648, 38.27253271 | Crust |
| Dachaidan lake | 18 | 95.26170015, 37.86029401 | Brine |
|  | 19 | 95.26170015, 37.86029401 | Crust |
| Xiaochaidan lake | 20 | 95.45797348, 37.47322326 | Brine |
|  | 21 | 95.45797348, 37.47322326 | Crust |
| Qarhan lake | 22 | 95.215430, 36.827310 | Brine |
|  | 23 | 95.416407, 37.022505 | Crust |
| Chaka lake | 24 | 99.078696, 36.731441 | Brine |
|  | 25 | 99.080787, 36.731546 | Crust |
| Keke lake | 26 | 98.223875, 36.971363 | Brine |
|  | 27 | 98.222874, 36.973189 | Crust |
| Qingtu lake | 28 | 103.62144169, 39.11021683 | Crust |
|  | 29 | 103.62115818, 39.10951652 | Brine |
| Yabulai | 30 | 102.73187559, 39.31245845 | Crust |
| Heihe | 31 | 100.08502299, 40.85021863 | Crust |
| Gurinai | 32 | 101.24538155, 40.70666235 | Crust |
|  | 33 | 101.24502777, 40.70577142 | Brine |
| West Juyan lake | 34 | 101.10208277, 42.29740063 | Saline-alkali soil |
| Swan lake | 35 | 101.59450158, 41.99856419 | Sand |
|  | 36 | 101.58832214, 41.99889558 | Saline-alkali soil |
| Jilantai | 37 | 105.72928168, 39.70082344 | Saline-alkali soil |
| Mu us | 38 | 107.57628812, 38.11149325 | Crust |

**Figure S1.** The δ^34^S values of different types of surface salts as a function of SO_4_/Na ratio in the Qaidam Basin, Alxa Plateau and Mu Us Desert. Red represents Qaidam, blue represents Alxa, and green represents Mu Us. Different sampling sites are distinguished by color depth.

**Figure S2.** The δ^34^S values of different types of surface salts as a function of SO_4_/Mg ratio in the Qaidam Basin, Alxa Plateau and Mu Us Desert. Red represents Qaidam, blue represents Alxa, and green represents Mu Us. Different sampling sites are distinguished by color depth.

**Figure S3.** The δ^34^S values of different types of surface salts as a function of SO_4_/total ratio in the Qaidam Basin, Alxa Plateau and Mu Us Desert. Red represents Qaidam, blue represents Alxa, and green represents Mu Us. Different sampling sites are distinguished by color depth.

**Figure S4.** Correlation analysis between δ^34^S values and various ion ratios (SO_4_/Na, SO_4_/Mg, and SO_4_/total ions) of salt samples in (a) Qaidam Basin, (b) Alxa Plateau. The “×” in the figure means that p＞0.05.
